# Effect of initial levothyroxine dose on neurodevelopmental and growth outcomes in children with congenital hypothyroidism

**DOI:** 10.3389/fendo.2022.923448

**Published:** 2022-09-05

**Authors:** Andrea Esposito, Maria Cristina Vigone, Miriam Polizzi, Malgorzata Gabriela Wasniewska, Alessandra Cassio, Alessandro Mussa, Roberto Gastaldi, Raffaella Di Mase, Gaia Vincenzi, Clara Pozzi, Elena Peroni, Carmela Bravaccio, Donatella Capalbo, Dario Bruzzese, Mariacarolina Salerno

**Affiliations:** ^1^ Pediatric Endocrinology Unit, Department of Translational Medical Sciences, University of Naples Federico II, Naples, Italy; ^2^ Endocrine Unit, Department of Pediatrics, IRCCS San Raffaele Hospital, Milan, Italy; ^3^ Pediatric Endocrinology Unit, Department of Mother and Child, University Hospital Federico II, Naples, Italy; ^4^ Department of Human Pathology of Adulthood and Childhood, University of Messina, Messina, Italy; ^5^ Unit of Pediatrics, University of Bologna, Bologna, Italy; ^6^ Pediatric Clinical Genetics Unit, Department of Public Health and Pediatrics, Regina Margherita Children Hospital, Torino, Italy; ^7^ Department of Pediatrics, IRCCS Istituto Giannina Gaslini, Genova, Italy; ^8^ Department of Pediatrics, Ospedale di Legnano, ASST Ovest milanese, Legnano, Italy; ^9^ Department of Public Health, University of Naples Federico II, Naples, Italy

**Keywords:** neonatal screening, congenital hypothyroidism, levothyroxine treatment, neurocognitive, growth

## Abstract

**Objectives:**

We designed a multicentre open prospective randomized trial to evaluate the risk-benefit profile of two different initial treatment schemes with levothyroxine (L-T4), 10-12.5 μg/kg/day vs 12.6-15 μg/kg/day, on growth and neurodevelopmental outcomes in children with congenital hypothyroidism (CH) detected by neonatal screening to identify the best range dose to achieve optimal neurocognitive development.

**Design, patients and methods:**

Children detected by neonatal screening were randomly assigned to receive an initial L-T4 dose of 10-12.5 μg/kg/day (Low) or 12.6-15 μg/kg/day (High). All patients underwent periodical clinical examination with measurement of growth parameters and measurement of TSH and FT4. Neurocognitive development was evaluated at the age of 24 months using Griffiths Mental Development Scales (GMDS) and cognitive and behavioral assessment was performed at 48 months of age using Wechsler Preschool and Primary scale of Intelligence (WIPPSI-III). The study was registered with clinicaltrials.gov (NCT05371262).

**Results:**

Treatment schemes below or above 12.5 μg/kg/day were both associated with rapid normalization of TSH and thyroid hormone levels in most patients with no differences in the risk of over- and under-treatment episodes in the first months of life. Growth parameters were normal and comparable between the two groups. Developmental quotients at 24 months of age were normal in both groups (Low 100.6 ± 15.5 vs High 96.9 ± 16.6). Likewise, at 4 years of age IQ and subtest scores were comparable between patients from Low and High (Total IQ 104.2 ± 11.4 vs 101.0 ± 20.3, Verbal IQ 103.9 ± 11.5 vs 98.7 ± 15.1, Performance IQ 105.3 ± 10.4 vs 100.3 ± 19.8). 6/45 CH patients (13.3%) showed a total IQ below 85 (73.7 ± 5.9) regardless of age at diagnosis, L-T4 starting dose, time of FT4 and TSH normalization and episodes of over and undertreatment. Worse socioeconomic status and delayed bone age at diagnosis were the only predictors of an increased risk of having suboptimal IQ at 24 and IQ at 48 months.

**Conclusions:**

Our results indicate that initial treatment with L-T4, 10-12.5 μg/kg/day vs 12.6-15 μg/kg/day, are both associated with normal growth and neurodevelopmental outcomes in children with CH detected by neonatal screening. Further studies with a long-term follow-up on a larger number of patients are needed to confirm these results.

**Clinical trial registration:**

https://clinicaltrials.gov/ct2/show/NCT05371262?term=NCT05371262&draw=2&rank=1 identifer NCT05371262.

## Introduction

Thyroid hormones are essential for nervous system development as they regulate several brain processes such as neuronal proliferation and migration, growth of axons and dendrites, myelination and synaptogenesis (1–3).

Congenital hypothyroidism (CH) is considered one of the most common preventable causes of intellectual disability (4).

Screening programs have led to early detection and treatment of infants with CH thus preventing the severe neurocognitive impairment resulting from late diagnosis (5) and allowing the achievement of normal adult height (6, 7).

Short- and long-term studies on neurocognitive function in early treated patients with CH have shown that, despite optimal cognitive development is achieved in the majority of CH patients, subtle neurocognitive deficits may still occur (8, 9).

Guidelines for CH recommend starting treatment with a dose of levothyroxine (L-T4) between 10 and 15 μg/kg/day, which has been associated with a faster normalization of both TSH and FT4 levels (10, 11). A recent systematic review suggests that only a starting dose >10 μg/kg/day is able to guarantee a normal neurocognitive outcome in patients with both severe and moderate CH (12). However, concern has been raised on the negative long-term effects of high initial doses of L-T4 on behavior and neurocognitive development due to the increased risk of overtreatment (13–17).

So far, only one randomized controlled study compared the effects of different L-T4 initial doses within this recommended range (18). Despite a few methodological limitations (19) the results of this study suggest that the highest is the initial dose in the treatment of CH the earliest is the achievement of euthyroid status and the best is the intellectual outcome (18, 20).

We designed this multicentre open prospective randomized trial to evaluate the risk-benefit profile of two different initial treatment schemes with L-T4, 10-12.5 μg/kg/day vs 12.6-15 μg/kg/day, on growth and neurodevelopmental outcomes in children with CH detected by neonatal screening to identify the best range dose to achieve optimal neurocognitive development.

Furthermore, secondary objective of this study was to evaluate the role of factors other than dose potentially influencing long-term growth and neurodevelopmental outcomes in children with CH.

## Materials and methods

### Study protocol

Six Italian centers were involved in this multicentre open prospective randomized trial. Patients were enrolled from May 2011 to May 2014. The study was registered with clinicaltrials.gov (NCT05371262).

In all subject recalled by neonatal screening, diagnosis of CH was confirmed by measurement of venous TSH, FT4 and FT3 together with measurement of thyroglobulin and thyroid autoantibodies.

Inclusion and exclusion criteria were defined in order to reduce the risk of enrolling subjects carrying factors potentially affecting growth and neurodevelopmental outcome, moreover a TSH cut-off greater than 30 mU/l was arbitrarily chosen to select patients who necessarily required treatment. Inclusion criteria were: age at diagnosis less than 30 days, Caucasian ethnicity and TSH at diagnosis above 30 mU/l. Exclusion criteria were: prematurity, major malformations, neonatal diseases, cromosomopathies and maternal thyroid disease.

Children with CH who fulfill both inclusion and exclusion criteria were enrolled and randomly assigned to receive an initial L-T4 dose of 10-12.5 μg/kg/day (Low) or 12.6-15 μg/kg/day (High). Randomization was designed according to a block scheme (8 blocks of 6 patients and 6 blocks of 4 patients which were randomly alternated) which guaranteed the frequency balance in the two groups during the enrolment without altering the causality of the assignment. Random allocation sequence was generated using the function *sample* in R statistical platform.

Bone maturation was assessed in all CH subjects at diagnosis, by evaluating the presence and the diameter of the epiphyseal nucleus of distal femoral at knee X-rays. Neonatal bone maturation, which is considered an indicator of intrauterine and severe CH, was considered delayed when the distal femur bony nucleus diameter was below 3 mm (21).

Thyroid morphology was assessed by thyroid ultrasound at diagnosis and by thyroid scan with either iodine-123 or technectium-99 at diagnosis or at the age of 36 months after L-T4 withdrawal.

Patients with eutopic gland, underwent diagnostic re-evaluation, at 3 years of age, after therapy withdrawal, to identify subjects with permanent vs transient CH.

### Clinical and biochemical evaluation

All patients underwent clinical examination and measurement of TSH and FT4 levels 7-10 days after the start of treatment and at 1.5, 3, 6, 9, 12, 18, 24, 30, 36, 42 and 48 months of life. Clinical evaluations included measurement of growth parameters (weight, length, cranial circumference) and the assessment of signs and symptoms of under- or over-treatment. Weight and length were expressed as standard deviation score (SDS) (22).

Initial L-T4 dose was modified, when necessary, in order to maintain serum TSH between 0.5 and 4.0 mU/l and serum FT4 in the upper normal range for age (1.4-2.3 ng/dl) (10). To evaluate the effects of under- or over-treatment during the follow-up on neurodevelopmental outcomes, we arbitrarily defined as index of under-treatment the number of episodes when serum TSH was >4.0 mIU/l and/or FT4 <1.4 ng/dl, and of over-treatment the number of episodes when serum FT4 was >2.3 ng/dl and/or TSH <0.4 mIU/l.

TSH and FT4 serum concentrations were measured by electrochemiluminescence immunoassay using a commercial kit (Roche Diagnostics) (reference ranges, TSH, 0.3-4.2 mIU/L; FT4, 0.75-1.7 ng/dL).

### Intellectual evaluation and socioeconomic status

Neurocognitive development in CH patients was evaluated at the age of 24 months using Griffiths Mental Development Scales (GMDS). The GMDS provides an overall developmental quotient (DQ) with subscales assessing skill areas: locomotor (subscale A), personal-social (subscale B), hearing and speech (subscale C), eye-hand coordination (subscale D) and performance (subscale E).

At 48 months of age cognitive and behavioral assessment was performed using Wechsler Preschool and Primary scale of Intelligence (WIPPSI-III). The WIPPSI-III evaluates the intelligence of children between 2.6 and 7.3 years and provides a Total Intelligence Quotient (TIQ), a Verbal Intelligence Quotient (VIQ), a Performance Intelligence Quotient (PIQ) and a Processing Speed Quotient (PSQ) for children from 4 years of life. WIPPSI-III for 4 years old children consist of 8 main subtests: Information, Vocabulary and Word Reasoning for VIQ; Block Design, Matrix Reasoning and Picture Concepts for PIQ; Symbol Search and Coding for PSQ.

All psychologists from different centers performed a training course in order to ensure homogeneous neurodevelopmental evaluations; moreover, they were blinded about the group the subjects belonged to.

The socioeconomic status was evaluated using the revised Graffar score (23), which distinguishes 5 socioeconomic levels according to the education and occupation of each parent, the main source of income of the family, and the home’s condition. A higher score indicates a lower socioeconomic status.

Informed consent was obtained from all parents and the study was approved by the Ethics Committee of University Hospital Federico II of Naples (protocol number 186/09).

### Outcomes

The primary outcome was the WIPPSI-III score for the Total Intelligence Quotient at 48 months. Secondary outcomes were the WIPPSI-III scores for Verbal Intelligence, Performance Intelligence and Processing Speed Quotients at 48 months and the GMDS, both DQ and subscales A, B, C, D and E at 24 months.

### Sample size

Sample size was estimated based on a clinically relevant between-groups difference in the WIPPSI-III Total Intelligence Quotient at 48 months equal to 5 points. Assuming a common standard deviation in the two populations equal to 5, a sample size of 27 children per treatment arms was deemed sufficient to detect such difference, if truly exists, with a two-sided significance level of 0.05 and a power of 0.80. Considering a drop-out rate of approximatively 20% and taking into account that about 5% of patients will have a transient form of CH and therefore will be excluded from the study, 72 children, 36 per treatment arm, was enrolled in order to have complete data for the primary end point analysis.

### Statistical analysis

We analyzed the primary endpoint and all secondary endpoints using the modified intention-to-treat (ITT) population defined as all randomized patients who received at least one dose of study treatment and for whom outcome data were available. For the cognitive endpoints, a sensitivity analysis was conducted in which missing outcome data were imputed using a multiple imputation approach. Variables entered in the imputation model were, besides treatment arm, age at diagnosis, Graffar score, severity of hypothyroidism, bone maturation and parameters of thyroid function at diagnosis (TSH and FT4). As the results of Multiple Imputation were practically equal to the complete-case analysis, only these were reported in the paper.

Demographic and clinical data referred to the baseline were summarized using standard descriptive statistics and compared between group (without reporting statistical significance) to assess whether good balance was achieved by randomization.

Primary and secondary end points were compared between groups using t-test for independent samples. Magnitude of effect was reported as mean difference with the corresponding 95% Confidence Intervals (95% CIs).

Longitudinal trajectories of thyroid function (TSH and FT4) and growth parameters (weight and height) during the follow-up period, were analyzed by using random-intercept linear mixed model (LMM) in which time from baseline was treated as categorical factor. Results of LMMs were reported as Estimated Marginal Means (EEMs) with the corresponding 95% CIs.

The exploratory analysis of potential predictors of long-term neurodevelopmental outcomes was based on univariable linear and logistic regression models, according to the numerical or dichotomic nature of the outcome variable. Results of these models are reported as mean differences and odds ratios (OR) with the corresponding 95% CIs.

All statistical analyses were conducted using the statistical platform R. *mice* package was used for multiple imputation.

## Results

One hundred twenty-five CH patients were assessed for enrolment in the study, 53 were excluded because did not fulfil all inclusion criteria or because parents refused to participate in the study. Thus, 72 patients were enrolled in the study and randomised in the two treatment groups.

**Figure 1:**
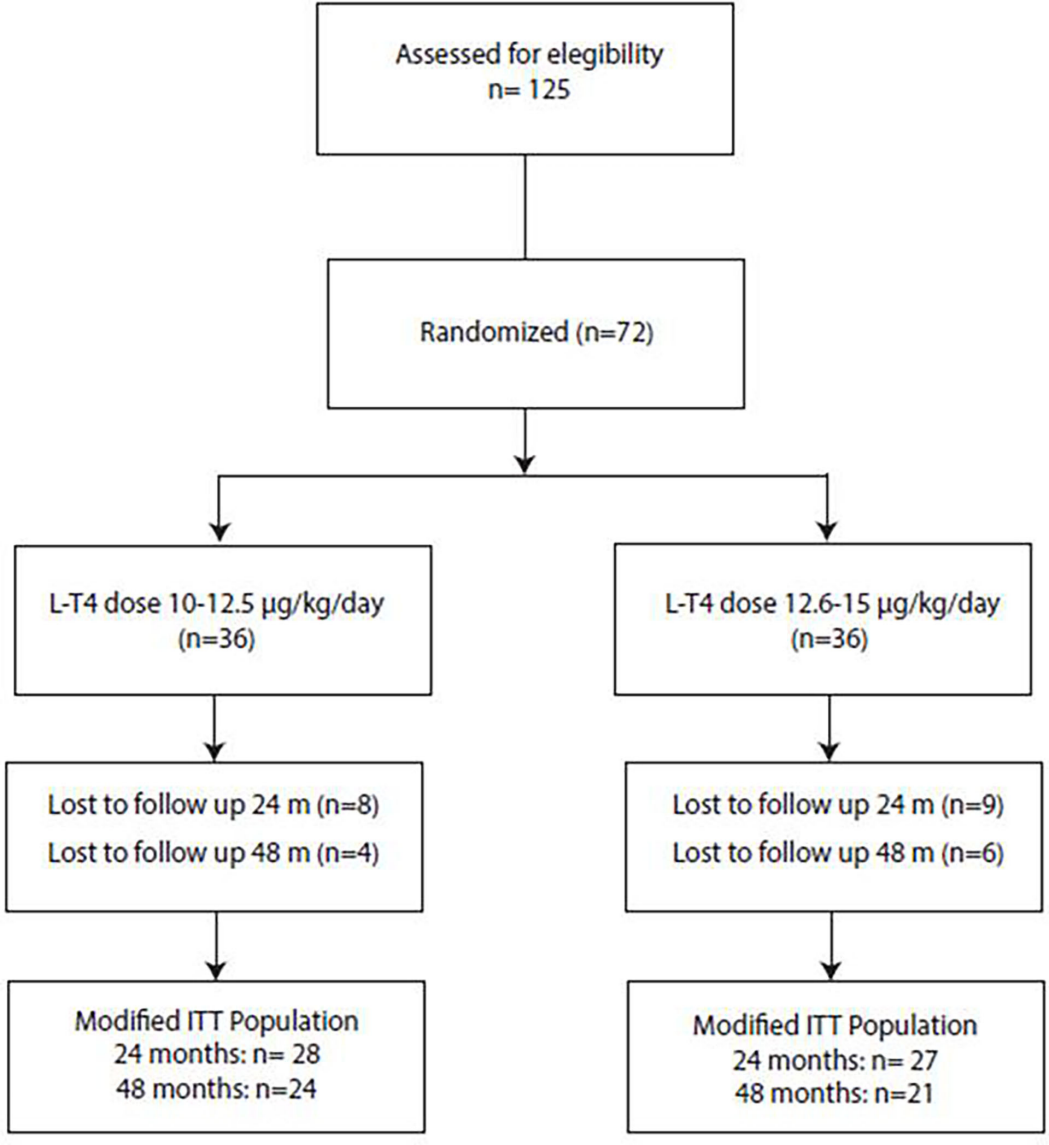
Flow-chart of the recruitment process. Of the 125 potentially recruitable patients with CH 53 were excluded because did not fulfil all inclusion and exclusion criteria or because parents refused to participate in the study. Thus, 72 patients were enrolled in the study and randomised in the two treatment groups. During the follow up period, 17 patients drop out from the study before 24 months of life and additional 10 were lost to follow up after 24 months. Thus, the modified ITT population consisted of 45 patients followed longitudinally in the first 4 years of life.

During the follow up period, twenty-seven (37.5%) patients dropped out from the study because of poor attendance at protocol schedule. The modified ITT population thus consisted of 45 patients followed longitudinally in the first 4 years of life (**Figure 1**).

Baseline characteristics of the patients divided in the two treatment groups 10-12.5 μg/kg/day vs 12.6-15 μg/kg/day at study entry are reported in **Table 1**. Overall, mean age at diagnosis was 13.53 ± 6.20 days. Severe hypothyroidism, defined as FT4 concentrations at diagnosis <0.4 ng/dl was observed in 34.8% of patients, with no differences between groups (36.4 vs 33.3%). The remaining 65.2% had moderate CH. Delayed bone maturation at the knee radiography was detected in 28% of patients. Thyroid scan revealed eutopic thyroid in 38.8% of patients and dysgenesis in the remaining 61.2%; in the latter group retrolingual ectopy was found in 40.3% and thyroid agenesis in 20.9% of patients. Overall, the two treatment groups were balanced, the only difference between the two groups was the L-T4 dose at baseline as expected based on the study design (**Table 1**).

**Table 1:** Baseline characteristics of patients randomized in the two groups of treatment at the enrollment in the study.

	Low (10-12.5 μg/kg/day)	High (12.6-15 μg/kg/day)
Number of patients	36	36
Sex Female/Male (%)	61.8/38.2	57.6/42.4
Gestational age (weeks)	39 (37-41)	40 (37-41)
Graffar Score	13 (4-18)	14 (4-18)
Age at diagnosis (days)	13.36 ± 5.55	13.71 ± 6.85
Moderate CH/Severe CH (%)	63.6/36.4	66.7/33.3
Adequate bone maturation/Retarded bone maturation (%)	80/20	64/36
Eutopic gland (%)	46.2	25
Ectopy gland (%)	38.5	45.8
Athyreosis (%)	15.4	29.2
TSH at diagnosis (mIU/l)	296.0 ± 235.0	341.2 ± 279.7
FT4 at diagnosis (ng/dl)	0.55 ± 0.31	0.53 ± 0.34
Initial L-T4 dose (μg/kg/day)	11.69 ± 0.65	13.47 ± 0.84

Data are expressed as percentage, median and range or mean ± standard deviation.

### Thyroid function

According to study protocol, patients were evaluated 7-10 days after starting L-T4 treatment (corresponding to a mean chronological age of 22.70 ± 6.25 days) and subsequently at 1.5 months of age (less than 1 month after the first post treatment evaluation).

**Figure 2:**
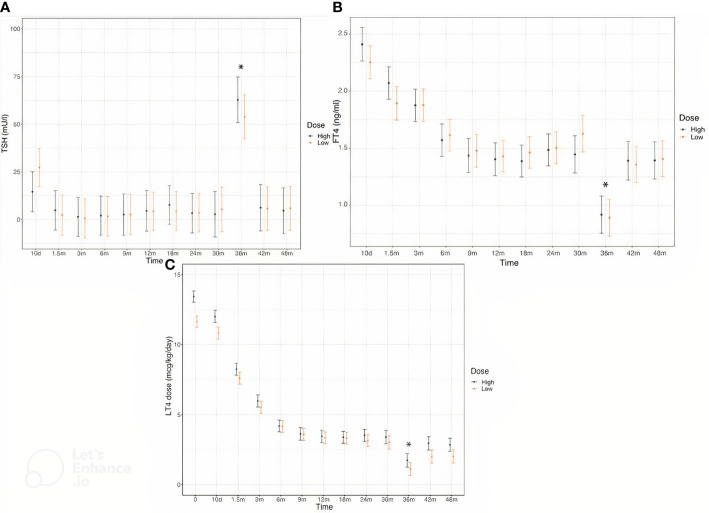
Serum concentrations of TSH **(A)**, FT4 **(B)** and L-T4 dose **(C)** in CH patients divided in the two treatment groups throughout the study. According to study protocol, thyroid hormones levels were evaluated at the enrollment, 10 days after L-T4 start and subsequently at the chronological age of 1.5, 3, 6, 9, 12, 18, 24, 30, 36, 42 and 48 months of life. * Changes in TSH and FT4 levels and in L-T4 dose at the age of 3 years are due to L-T4 withdrawal in the majority of patients for the re-evaluation thyroid function.

Serum concentrations of TSH and FT4 were comparable in the two groups throughout the follow-up (**Figures 2A, B**). L-T4 dose was higher in High at baseline and remained significantly different in the first 7-10 days after therapy initiation, by the age of 1.5 months this difference was no longer appreciable (**Figure 2C**). The frequency of dose adjustment during the first six months of treatment as well as in the subsequent months was similar in both groups.

No differences in the number of episodes of over- and under-treatment during the first 6 months of therapy were recorded in the two groups of patients.

### Growth

**Figure 3:**
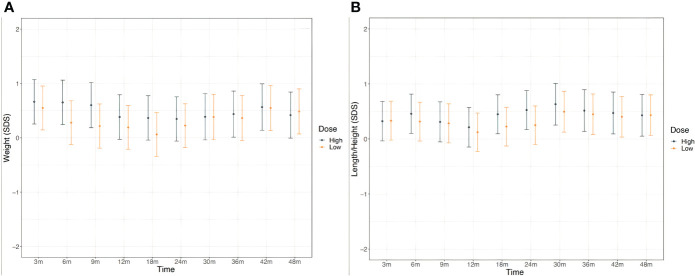
Weight **(A)** and height **(B)** in CH patients in the two treatment groups throughout the study. According to study protocol, enrolled patients underwent clinical evaluation at the enrollment, 10 days after L-T4 start and subsequently at the chronological age of 1.5, 3, 6, 9, 12, 18, 24, 30, 36, 42 and 48 months of life.

Weight and length/height were normal and comparable between the two treatments groups all over the study period as depicted in **Figure 3**.

### Neurocognitive development

All patients were euthyroid at the time of neurocognitive evaluations. Overall, at 24 months of age mean developmental quotients (DQ 98.80 ± 15.98) and subscale scores were normal (Subscale A 110.64 ± 22.22, Subscale B 96.98 ± 23.17, Subscale C 89.18 ± 20.57, Subscale D 103.05 ± 15.66, Subscale E 103.71 ± 15.02). No significant differences were observed when CH patients were divided on the basis of the initial treatment regimen both in the modified ITT population (**Table 2**).

**Table 2:** Developmental Quotients and subscale scores at 24 months of age and Intelligence Quotients and subtest scores at 48 months of age in the two groups of patients with different initial treatment regimen.

	Low (10-12.5 μg/kg/day)	High (12.6-15 μg/kg/day)	Between-Group Difference (95% CI)	P
**Age 24 months**				
Number of patients	28	27		
Developmental quotient	100.6 ± 15.5	96.9 ± 16.6	3.8 (-4.9 to 12.4)	0.39
Subscale A	112.1 ± 21.4	109.1 ± 23.3	3 (-9.1 to 15.1)	0.62
Subscale B	100.5 ± 20.7	93.3 ± 25.3	7.2 (-5.3 to 19.7)	0.26
Subscale C	90.6 ± 19.7	87.7 ± 21.7	2.8 (-8.4 to 14)	0.61
Subscale D	105.4 ± 13.5	100.7 ± 17.5	4.7 (-3.8 to 13.1)	0.27
Subscale E	105.0 ± 14.9	102.4 ± 15.4	2.6 (-5.6 to 10.7)	0.53
**Age 48 months**				
Number	24	21		
Total Intelligence Quotient	104.2 ± 11.4	101.0 ± 20.3	3.1 (-6.6 to 12.8)	0.54
Verbal Intelligence Quotient	103.9 ± 11.5	98.7 ± 15.1	5.2 (-2.8 to 13.3)	0.20
Performance Intelligence Quotient	105.3 ± 10.4	100.3 ± 19.8	5 (-4.3 to 14.3)	0.31
Processing Speed Quotient	99.2 ± 13.1	97.1 ± 18.3	2.1 (-7.4 to 11.5)	0.67
Information	10.2 ± 2.3	9.1 ± 3.3	1.1 (-0.6 to 2.8)	0.21
Vocabulary	11.5 ± 2.4	10.2 ± 2.6	1.3 (-0.3 to 2.8)	0.10
Word Reasoning	10.3 ± 3.1	10.1 ± 2.8	0.2 (-1.6 to 2)	0.82
Block Design	9.7 ± 2.2	8.6 ± 3.6	1.1 (-0.6 to 2.9)	0.22
Matrix Reasoning	10.7 ± 2.6	11.6 ± 3.6	-0.9 (-2.8 to 1)	0.35
Picture Concepts	11.5 ± 2.8	10.0 ± 3.2	1.5 (-0.2 to 3.2)	0.10
Symbol Search	10.6 ± 2.6	9.5 ± 3.4	1.1 (-0.7 to 3)	0.22
Coding	9.1 ± 2.5	9.6 ± 3.7	-0.5 (-2.4 to 1.4)	0.60

Data are expressed as mean ± standard deviation.

Similarly, at 4 years of age CH patients had normal Intelligence Quotient scores (TIQ 102.71 ± 16.02, VIQ 101.47 ± 13.41, PIQ 103.00 ± 15.51, PSQ 98.24 ± 15.59). IQ and subtest scores at 48 months of age were comparable between patients from Low and High (**Table 2**).

Six out of 45 CH patients (13.3%) showed a total IQ below 85 (73.7 ± 5.9) and of them only 1 had an IQ below 70 (2%). In details, mean QIV was 79.7 ± 7.5, mean QIP was 73.2 ± 6.7, mean PSQ was 74.3 ± 5.5. Five out of 6 patients were treated with a L-T4 initial dose above 12.6 μg/kg/day, 3/6 had athyreosis with severe CH and delayed bone age at diagnosis, 1/6 had ectopic and 2/6 eutopic gland and all had high Graffar score indicating a low socioeconomic status. 5/6 patients experienced 2-4 episodes of over-treatment while the remaining 1 experienced 3 episodes of under-treatment within the first year of treatment.

**Table 3** summarizes the impact of socioeconomic status and bone age retardation on neurocognitive outcome. A unit increase in Graffar score (which means a worsening of socioeconomic status) was associated with an increased risk of having suboptimal DQ at 24 and IQ at 48 months. Conversely, a unit increase in the diameter of the distal femoral epiphyseal nucleus (which means a less severe CH) was associated with a reduced risk of suboptimal DQ and IQ.

**Table 3:** Impact of Graffar score and femoral nucleus diameter on long-term neurodevelopmental outcomes.

	Graffar score	Femoral nucleus diameter
**24 months**		
Developmental quotient	**1.39 (1.07 to 2.02) p=0.04**	0.8 (0.56 to 1.12) p=0.2
Subscale A	1.1 (0.89 to 1.42) p=0.43	**0.62 (0.39 to 0.91) p=0.02**
Subscale B	**1.34 (1.1 to 1.73) p=0.01**	**0.71 (0.52 to 0.94) p=0.02**
Subscale C	1.05 (0.91 to 1.21) p=0.54	0.98 (0.77 to 1.26) p=0.89
Subscale D	**1.57 (1.11 to 2.64) p=0.04**	0.75 (0.49 to 1.1) p=0.16
Subscale E	1.18 (0.94 to 1.61) p=0.2	1.06 (0.72 to 1.62) p=0.77
**48 months**		
Total Intelligence Quotient	**1.51 (1.07 to 2.54) p=0.05**	**0.62 (0.38 to 0.92) p=0.03**
Verbal Intelligence Quotient	1.26 (0.99 to 1.74) p=0.1	0.8 (0.54 to 1.13) p=0.21
Performance Intelligence Quotient	**1.51 (1.07 to 2.54) p=0.05**	**0.62 (0.38 to 0.92) p=0.03**
Processing Speed Quotient	**1.72 (1.19 to 2.95) p=0.02**	**0.66 (0.43 to 0.93) p=0.03**

Data are expressed as Odds Ratio for having a long-term neurodevelopmental outcome lower than clinical threshold (<85 point) for every unit increase in Graffar score or femoral nucleus. Results were obtained using univariable logistic regression models. Bold values has been used to underline the results but it is not necessary.

The presence of at least one episode of under-treatment in the first six months of life was associated with higher risk of impaired locomotor outcome at 24 months of live (OR: 5.45, 95% CI: 1.16 to 30.25, p=0.036); the higher the number of the episodes the lower was locomotor quotient at 24 months (mean difference for each incremental episode: -7.8, 95% CI: -15.3 to -0.3, p=0.041).

Conversely, the presence of at least one episode of FT4 levels above range in the first six months of life were associated with reduced risk of impaired verbal outcome at 48 months of life (OR: 0.18, 95% CI: 0.02 to 0.91, p=0.042) and the higher the number of over-treatment episodes the higher was VIQ at 48 months (mean difference for each incremental episode: +8, 95% CI: 2.3-13.6, p=0.007). Moreover, after six months of age episodes of over- or under- treatment were not correlated with neurocognitive outcomes

Overall, the number of over/under-treatment episodes were independent of the L-T4 treatment group the belonged to. In particular, episodes of overtreatment at 12 months were 2.1 ± 1.4 vs 1.8 ± 1.6 (p=0.467) and episodes of undertreatment were 1.1 ± 1.3 vs 0.8 ± 1.1 (p=0.307), in Low vs High respectively.

Multivariable linear and logistic regression models did not reveal significant correlations between severity or etiology of CH and neurocognitive outcomes.

## Discussion

The improvement of neonatal screening programs and early treatment with a high initial L-T4 above 10 μg/kg per day has resulted in normal neurodevelopmental outcomes in children and young adults with CH (4) even compared with sibling controls (12, 24).

However, concern has been raised on the negative long-term effects of high initial doses of L-T4 on behavior and neurocognitive development due to the increased risk of overtreatment (13–17).

The results of this multicenter randomized study indicate that different L-T4 starting regimens, within the range of 10-15 μg/kg/day, have comparable effects on growth and neurocognitive outcomes during the first four years of life in children with CH detected by neonatal screening irrespectively from the severity of hypothyroidism. Indeed, treatment schemes below or above 12.5 μg/kg/day were both associated with rapid normalization of TSH and thyroid hormone levels in most patients with no differences in the risk of over- and under-treatment episodes in the first months of life.

In the study by Albert et al. patients were treated from a mean age of nine days with a L-T4 starting dose between 10-15 μg/kg depending on CH severity. No gap was observed comparing 44 CH patients and 53 unaffected sibling controls (24).

In another study from Aleksander et al. the neurocognitive outcome of 76 young adults with CH with a mean age of 18.1 years was compared with 40 sibling controls with a mean age of 19.8 years (12). The median age at diagnosis was eight days and the mean L-T4 starting dose was 13.5 μg/kg per day. There was no difference in overall IQ nor differences in attention, memory, fine motor skills, quality of life scores.

In both studies TSH normalized within a median time of 15 days after diagnosis.

Moreover, in a meta-analysis included in the latter study comparing IQ differences between severe and mild CH cases with respect to the starting dose revealed that even children with severe CH can reach a normal IQ if L-T4 treatment is started with a dose of at least 10 μg/kg (12).

However, a few patients with severe CH may still have subtle cognitive and motor deficits, and lower educational attainment despite early treatment with a high starting L-T4 dose (25, 26).

Recently, Perri et al. evaluated 28 children with permanent CH at a mean age of 9 years. Mean IQ was normal and comparable to controls however, there was a great variability in IQ values with a high percentage of CH patients having sub-optimal IQ (28.6%) and intellectual disability (10.7%). A significant impairment was detected in specific neurocognitive outcomes such as processing speed, visual attention, reading skills and arithmetic which correlated with white matter structure abnormalities (9). In this study the age at the beginning of treatment was quite variable (15.3 ± 7.9 days) as well as L-T4 starting dose (9.46 ± 2.28 µg/kg/day) and the mean TSH values (6.98 ± 4.68 µU/L) at the time of cognitive assessment was mildly elevated in some CH patients.

In our study, all patients were euthyroid at the time of neurocognitive evaluation and mean values of both developmental quotient at 24 months and intelligence quotient at 48 months of age were normal with no differences in specific skills. The percentage of patients with subnormal IQ (13.3%) was quite lower and was independent from age at diagnosis, L-T4 treatment dose and the time required to normalize thyroid function. However, the finding of CH patients with subnormal IQ suggest that neurodevelopmental rescue should not be taken for granted even in the era of neonatal screening (27). Prenatal brain damage due to thyroid hormone insufficiency *in utero*, may not be completely prevented by trans-placental supply of maternal thyroxine and may not be completely reverted by postnatal treatment (9, 28).

On the other hand, overtreatment can also affect neurodevelopmental outcome.

High-dose treatment has been associated with an increased risk of episodes of overtreatment in the first months postnatally, a critical period for brain development, with adverse cognitive outcome in some children with CH (14, 29, 30). In addition, a recent study, in a limited number of CH patients evaluated at 6 and 11 years of age, suggested that over-treatment in the first 1-3 months of life might be associated with attention deficit hyperactivity syndrome whereas under-treatment in the first 3-6 months with behavioral problems indicative for autism (16).

In our study, the number of over and under-treatment episodes in the first 6 months of treatment was independent of the L-T4 treatment regimen and was not associated with adverse neurocognitive outcome at 4 years of age.

To date only one randomized controlled study compared the effects of different L-T4 initial doses within the range of 10-15 μg/kg/day on neurocognitive development reporting higher intelligent quotient in CH children treated with a mean initial dose of 14.5 μg/kg/day compared with children treated with a mean initial dose of 10.9 μg/kg/day (20). However, this study had several limitations such as an unclear randomization and treatment scheme, small sample size and great variability of the age at neurocognitive evaluation.

The strength of our study is that it has been well designed in terms of randomization and that included a large population of children ensuring appropriate evaluation of any differences depending on different doses of L-T4. Moreover, all CH patients received the same protocol of neurocognitive evaluation at the same age. We acknowledge that a major limitation of our study is a sample size of the modified ITT population slightly smaller than that anticipated in the power analysis and a variability of the main outcome measure larger than expected. These limitations could have determined a loss of power that could partly account for the non-significant results. Moreover, another limitation is the short duration of the follow-up, indeed subtle deficits in specific cognitive domains may become detectable at older ages. Finally, thyroid agenesis and ectopic CH may affect the results of the analysis when analyzed together with eutopic CH, as higher doses of L-T4 are generally required in patients with thyroid agenesis and ectopic CH and these disease groups may differ from eutopic CH in the level of hypothyroidism in the fetal period. Actually, the randomized design of the study, did not allow a separated analysis of data based on different etiologies.

The present study highlights that worse socioeconomic status and delayed bone age at diagnosis were the only predictors of an increased risk of having suboptimal IQ at 24 and IQ at 48 months. In agreement with our findings, other studies have documented a close association between higher social class and either better IQ or better academic achievement (25, 31–34).

Socioeconomic status is an important predictor of neurocognitive performance (35). Indeed, children with lower socioeconomic status are more likely to have worse cognitive abilities (36) and to obtain lower scores on standardized tests of academic achievement (37). Furthermore, a large Korean population-based cohort study reported a significant combined effect for low family income and neonatal hypothyroidism on the risk of intellectual disability in children (38).

Bone maturation at birth has been proposed as marker of prenatal hypothyroidism severity and has been associated with slight neurocognitive deficits (21, 26, 39).

Finally, early detection and treatment of CH are also important to linear growth, onset and progression of puberty, and final height attainment of CH patients (6, 7, 40). In this study all patients showed normal weight and length/height growth regardless of the initial L-T4 starting dose.

In conclusion our results indicate that initial treatment schemes with L-T4, 10-12.5 μg/kg/day vs 12.6-15 μg/kg/day, are both associated with normal growth and neurodevelopmental outcomes in children with CH detected by neonatal screening; however, further studies with a long-term follow-up on a larger number of patients are needed to confirm these results.

## Data availability statement

The raw data supporting the conclusions of this article will be made available by the authors, after justified request.

## Ethics statement

The study was reviewed and approved by Ethics Committee of University Hospital Federico II of Naples. Written informed consent to participate in this study was provided by the participants’ legal guardian/next of kin.

## Author contributions

AE has contributed in following the patients, collecting the data, drafting, and revising the manuscript. MCV, MGW, AC, AM, RG, GV, EP have participated to the multicenter study by enrolling and taking care of their patients as well as by revising the manuscript. RDM and DC take care of the patients and revised the manuscript. MP, CP, CB take care of the neurocognitive outcome of the patients and revised the manuscript. DB designed the randomization of the study and performed statistical analysis. MS designed the study, supervised the patients and actively participated in data analysis, drafting and revision of the manuscript. All authors approved the final manuscript as submitted and agree to be accountable for all aspects of the work.

## Funding

This work was supported by a grant from the Italian Medicines Agency (AIFA) Grant No. FARM8A8FHP to MS.

## Conflict of interest

The authors declare that the research was conducted in the absence of any commercial or financial relationships that could be construed as a potential conflict of interest.

## Publisher’s note

All claims expressed in this article are solely those of the authors and do not necessarily represent those of their affiliated organizations, or those of the publisher, the editors and the reviewers. Any product that may be evaluated in this article, or claim that may be made by its manufacturer, is not guaranteed or endorsed by the publisher.
